# Association Between Arterial Stiffness Index and Age-Related Diseases: A Mendelian Randomization Study

**DOI:** 10.1089/rej.2024.0041

**Published:** 2025-01-28

**Authors:** Xiaojie Yu, Yang Cao, Xinyi Li, Qingchun Liang, Xiaodan Dong, Bing Liang

**Affiliations:** ^1^ Department of Anesthesiology, Guangzhou Red Cross Hospital, Jinan University, Guangzhou, China.; ^2^ Department of Anesthesiology, The Third Affiliated Hospital, Southern Medical University, Guangzhou, China.

**Keywords:** age-related disease, arterial stiffness index, Mendelian randomization analysis, atherosclerosis

## Abstract

Arterial stiffness is an emerging indicator of cardiovascular risk, but its causal relationship with a variety of age-related diseases is unclear. The objective is to assess the causal relationship between arterial stiffness index (ASI) and age-related diseases by Mendelian randomization (MR) analysis. We obtained instrumental variables associated with age-related diseases from genome-wide association studies (GWAS) of 484,598 European individuals, and data for ASI were obtained from the UK Biobank GWAS of 127,127 participants. We used the inverse variance-weighted as the primary analysis method. In addition, several sensitivity analyses including MR-Egger, weighted-median (WM), Mendelian randomization pleiotropy residual sum and outlier, and Cochran’s Q test were performed to test the robustness of the results. Reverse MR analysis was also performed to assess reverse causal relationships between age-related diseases and ASI. We verified the causal relationship between eight age-related diseases and ASI, of which cardiovascular disease (*β* = 0.19), gallbladder disease (*β* = 0.85), liver, biliary, or pancreas problem (*β* = 1.02), hypertension (*β* = 0.19), joint disorder (*β* = 0.53), and esophageal disorder (*β* = 2.10) elevated ASI. In contrast, hyperthyroidism or thyrotoxicosis (*β* = −2.17) and bowel problems (*β* = −1.83) may reduce ASI. This MR analysis reveals causal relationships between ASI and several age-related diseases. ASI is expected to be a potential indicator of health conditions for older populations.

## Introduction

As the global aging process intensifies, health issues of the aged have become the focus of worldwide concern. It is estimated that by 2030, there will be more than one billion seniors over the age of 65 across the globe.^1^ Aging brings not only huge economic burdens to society but also challenges to the health care system. According to statistics, health care expenditures for old persons take up more than 50% of the total health spending of a family. A survey in China found that, in 2015, health care spending for the very unhealthy elderly population was $26.112 billion, compared with only $5.542 billion for the healthy elderly population.^2^ It is clear that keeping older people healthy is essential to ease the pressure on society’s health care expenditure.

Aging is an irreversible biological process. As individuals age, they become more susceptible to various diseases and organ damage. It is widely believed that this is due to the accumulation of damage caused by different stress factors.^3^ However, with the development of human genome research, studies have found that many diseases are linked to aging genes. Dönertaş et al. clustered data on age-related diseases from the UK Biobank (UKBB) and found high levels of genetic similarity within clusters of diseases with similar age-of-onset profiles.^4^ By using AI-powered PandaOmics platform, Pun et al. predicted 484 genes associated with age-related diseases based on analyzing multiple data sources such as literature and omics.^5^ Aging-related diseases, including cardiovascular diseases (CVDs), endocrine diseases, and gastrointestinal diseases, are mainly threatening the health of older people. Research on age-related genes and related biomarkers helps to comprehend disease mechanisms and improve the life quality of old people.

As an emerging biomarker for CVD risk detection, arterial stiffness could be a potential indicator in screening health conditions in the aged. Pulse wave velocity (PWV) is currently the common indicator and gold standard for measuring arterial stiffness, higher PWV has been proven to be directly associated with CVD.^6,7,54^ Based on photoplethysmography, the arterial stiffness index (ASI) is another more convenient and inexpensive noninvasive method of assessing arterial stiffness.^8^ The subject could take a seated position and by clipping the infrared sensor to a finger for 10–15 seconds the pulse waveforms were obtained. The first section of the waveform records the transmission of pressure from the left ventricle to the finger, while the second section indicates the transmission of pressure to the lower body via the aorta. The height of the subject (meters) divided by the time between peaks in the bidirectional waveform (meters per second) gives the ASI.^9^ It has been verified by three independent studies that the assessment of arterial stiffness by ASI is consistent with PWV.^10^ Higher ASI values not only indicate a stiffer arterial wall but may also accelerate the progression of diseases in other systems.

Actually, the causal relationship between age-related diseases and ASI remains unclear. In this study, we used Mendelian randomization (MR) to assess the causal relationship between outcome and exposure. MR analysis is a popular epidemiological method for inferring causality, by using genetic variants from the genome-wide association studies (GWAS) databases as instrumental variables (IVs). The well-known Mendelian law describes that parental alleles are randomly assigned to offspring, so an MR study is regarded as a “natural” randomized controlled trial.^11^ Since genetic variants are present from birth, the effects of acquired environmental confounders can be avoided. With the development of genomic technologies, MR studies have been widely used in studies of causal relationships between diseases and biomarkers or health indicators.^12–14^

## Materials and Methods

### Study design

**FIG. 1.**
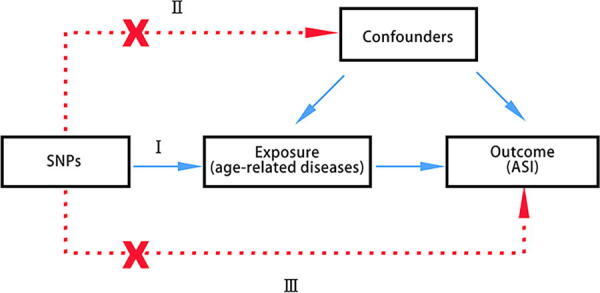
Three assumptions of Mendelian randomization study. (I) Instrumental variables (IVs) are highly linked to the exposure; (II) IVs are not confounders related; and (III) IVs affect outcome only through exposure. ASI, arterial stiffness index; SNPs, single-nucleotide polymorphisms.

There are three assumptions in the basic principles of MR: (1) IVs are highly linked to exposure; (2) IVs are not confounders related; and (3) IVs affect outcome only through exposure. The design of the study is illustrated in Figure 1.

### Data sources

We obtained single nucleotide polymorphisms (SNPs) from publicly available GWAS. The 116 age-related diseases in this study were taken from research by Dönertaş et al.^4^ on common genetic associations between age-related diseases. The genetic variables of these self-reported diseases were from UKBB, included 484,598 participants under 70 years old. The data of ASI were from the latest GWAS of 127,121 participants.^15^ In order to minimize population stratification bias, all subjects included in our study were of European ancestry.

### SNP selection

First, instrumental variables had a *p*-value of <5 × 10^−8^ to be strongly associated with exposure, thus SNPs associated with age-related diseases were selected based on a genome-wide significance threshold of *p* < 5 × 10^−8^. Second, SNPs with linkage disequilibrium (LD) were removed, setting the LD clumping thresholds as *r*^2^ < 0.001 and clumping window >10,000 kb, which means removing SNPs with *r*^2^ > 0.001 within 10,000 kb from the significantly associated SNPs. Lastly, we harmonized the SNPs of exposure and outcome in order to eliminate palindromic SNPs.

### MR analysis

We used three MR methods to assess the causal associations, includes inverse variance-weighted (IVW), MR-Egger, and weighted-median (WM). The IVW was the primary method because it gives the most accurate and stable results when there is no pleiotropy, the two other methods provide complementary evidence for the results of IVW. According to the principles of the Bonferroni correction, since 116 two-sample MR analyses were performed in this study, setting *p* < 0.05/116 ≈ 4.31 × 10^−4^ was statistically significant when 4.31 × 10^−4^ < *p* < 0.05 indicated that the result was suggestive, whereas *p* ≥ 0.05 was considered negative. We also examined heterogeneity by Cochran’s Q test,^16^ with *p*-values <0.05 indicating the results are heterogeneous. Pleiotropy was assessed by the intercept value of MR-Egger regression,^17^ which approaches zero meaning no directional pleiotropy (*p* > 0.05).^18^ And the Mendelian randomization pleiotropy residual sum and outlier (MR-PRESSO) was used to identify outliers. Leave-one-out analysis examined the effect of remaining instrumental variables on the results by excluding each SNP to find out if there were individual SNPs exerting a significant effect on the results. Additionally, we performed reverse MR analysis to estimate the effect of ASI on age-related diseases as well. All analyses were conducted using the latest version of R (4.3.2) with TwoSampleMR package and MR-PRESSO package.

## Results

**FIG. 2.**
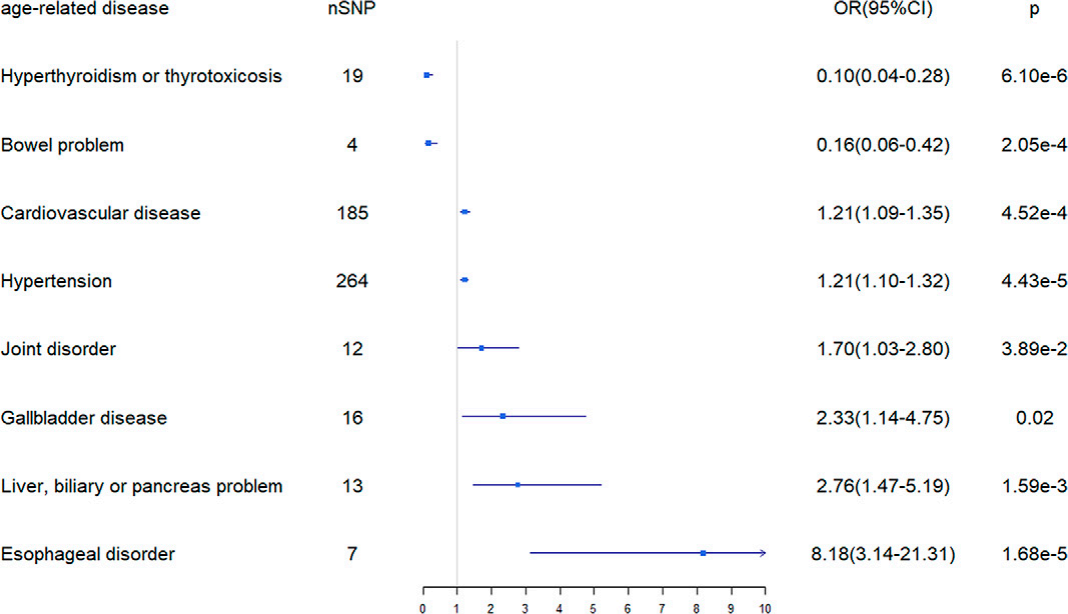
MR analysis of eight age-related diseases with ASI by IVW method. nSNP, number of single-nucleotide polymorphisms; OR, odds ratio; CI, confidence interval; ASI, arterial stiffness index.

Among the 116 diseases, we excluded the diseases with one SNP only and conducted MR analysis for the remaining diseases. The IVW method showed that eight diseases have causal relationships with ASI. CVD (*β* = 0.19 [95% CI, 1.09–1.35]), gallbladder disease (*β* = 0.84 [95% CI, 1.14–4.75]), liver, biliary, or pancreas problem (*β* = 1.02 [95% CI, 1.47–5.19]), hypertension (*β* = 0.19 [95% CI, 1.10–1.32]), joint disorder (*β* = 0.53 [95% CI, 1.03–2.80]), and esophageal disorder (*β* = 2.10 [95% CI, 3.14–21.31]) were positively associated with ASI; meanwhile, hyperthyroidism or thyrotoxicosis (*β* = −2.27 [95% CI, 0.04–0.28]) and bowel problem (*β* = −1.83 [95% CI, 0.06–0.42]) led to a lower ASI (Fig. 2). The full MR results are available in Supplementary Table S3.

The Cochran’s Q test showed no evidence of heterogeneity except for CVDs (*p* = 6.97e-09) and hypertension (*p* = 2.63e-10). So, we adopted a random-effects model to eliminate heterogeneity. MR-Egger regression suggested no directed pleiotropy and MR-PRESSO indicated no outliers, as all the analyses have a *p*-value >0.05. The total sensitivity analysis results are shown in Table 1. Scatter plot, funnel plot, and forest plot of the association between these eight age-related diseases and ASI also supported the above results (Supplementary Figures S1, Supplementary Figure S2 and Supplementary Figure S3), the leave-one-out analysis showed no individual SNP significantly affected the results (Supplementary Figure S4). In the reverse MR, all results of the IVW method indicated no significant causal association with eight diseases when ASI acted as exposure (Supplementary Table S4).

**Table 1.** Summary of Sensitivity Analysis Results for the Eight Diseases

Exposure	Cochran’s Q test (*p*-value)		MR-Egger intercept (*p*-value)	MR-PRESSO (*p*-value)
	MR-Egger	IVW		
Bowel problem	0.30	0.38	0.52	0.668
Gallbladder disease	0.30	0.22	0.21	0.263
Liver, biliary, or pancreas problem	0.50	0.55	0.57	0.413
Hypertension	3.39e-10	2.63e-10	0.21	0.369
Joint disorder	0.38	0.37	0.31	0.358
Hyperthyroidism or thyrotoxicosis	0.61	0.52	0.15	0.563
Esophageal disorder	0.52	0.39	0.21	0.197
Cardiovascular disease	5.40e-09	6.97e-09	0.80	0.816

IVW, inverse variance-weighted; MR, Mendelian randomization; MR-PRESSO, MR pleiotropy residual sum and outlier.

## Discussion

In this MR study, we evaluated the association between 116 age-related diseases and the indicator of arterial stiffness, ASI. The results revealed that CVD, hypertension, joint disease, gallbladder disease, esophageal disease, and liver, biliary, or pancreas problem increased ASI, whereas hyperthyroidism or thyrotoxicosis and bowel problems reduced ASI.

Several limitations should be recognized in this study. First, directional pleiotropy is inevitable in MR studies. Second, all of the GWAS databases in this study were from European ethnic groups, this limits the generalizability of the findings to a broader population. Third, compared with ASI, which assesses arterial stiffness through changes in arterial volume, PWV is a more direct measure of stiffness. However, there are no adequate GWAS databases of PWV present, and there is a lack of standardization as PWV can be measured in different parts of the body. Some studies have suggested poor agreement between ASI and PWV in severe hypertensive populations,^19^ the difference in measurement methods may lead to inconsistency of results in the analyses.

### Cardiovascular disease

Most cardiac diseases originate from coronary atherosclerosis. In the United States, coronary artery disease accounts for 64% of all cardiac deaths,^20^ while 30% of all deaths are related to atherosclerotic cardiovascular disease (ASCVD) on a world scale.^21^ A comparative study found that patients with CAD had significantly higher brachial-ankle PWV than those without CAD. Another meta-analysis also indicated that greater carotid artery stiffness was associated with higher total cardiovascular event incidence and higher total cardiovascular and all-cause mortality.^22^ Given that there are different measurements of arterial stiffness, a cohort including 579 samples with an average age of 67 years, which assessed arterial compliance by ultrasonography, carotid-femoral PWV, and aortic augmentation index, respectively, showed that carotid and femoral arterial stiffness were independently associated with cardiovascular events and all-cause mortality.^23^

ApoB carries LDL cholesterol into the arterial wall and, after oxidation, it is phagocytosed by macrophages, generating foam cells and thus leading to atherosclerosis. However, cholesterol can efflux from arterial wall foam cells to form HDL cholesterol particles; this process is called macrophage reverse cholesterol transport.^55^ However measuring LDL or HDL levels alone to assess cardiovascular risk is insufficient. In contrast, ASI calculates the ratio of beneficial and harmful lipoproteins in the body, providing a more comprehensive indicator.

### Gallbladder disease

Gallbladder diseases encompass cholecystitis, gallstone disease, gallbladder polyps, gallbladder cancer, and others. Among these, cholecystitis with gallstones is the most common. In the United States, about 20 million individuals suffer from gallbladder disease.^24^ And the prevalence of gallbladder disease is increasing every year in developed countries. Gallstone disease, for example, has risen to 13.9% in the United States since 2020, and the incidences of gallbladder disease are rising with age.^25^ Also, it was found that most of the patients with gallbladder disease have obesity, hyperlipidemia, diabetes, hypertension, and other metabolic syndromes.^56^

An investigative follow-up on 5209 Framingham Heart Study volunteers discovered that the risk of subsequent coronary disease was increased in men with cholelithiasis but not significantly in women patients.^26^ Further studies are still needed to prove the relationship between cholelithiasis and CVD.

### Liver, biliary, or pancreas problem

Nonalcoholic fatty liver disease (NAFLD) is the most common cause of chronic liver disease in developed countries. Much evidence suggests that NAFLD is a multisystem disease that affects multiple organs, especially the cardiovascular system. Mortality in patients with NAFLD is predominant due to cardiovascular disease rather than liver disease *per se*.^27^ A cohort study including 229 patients with biopsy-confirmed NAFLD found a significantly increased risk of cardiovascular death in patients with NAFLD after up to 33 years of follow-up.^28^ Moreover, cross-sectional studies have found widespread manifestations of atherosclerosis such as coronary artery calcification, increased carotid intima-media thickness, and impaired flow-mediated vasodilation in patients with NAFLD.^29^ In a cohort study of 755 healthy adult men by Moon et al., a strong and independent association between NAFLD and carotid artery inflammation was found as assessed by 18F-fluorodeoxyglucose positron emission tomography.^30^ Therefore, targeted screening of populations with liver disease by using indicators such as ASI may be able to assess cardiovascular disease risk.

### Hypertension

Blood pressure is known to increase as we age. As the global population ages, hypertension will become more and more common. According to the latest NCHS data, the prevalence of hypertension in the United States among adults aged 18 years and over was 45.4% in 2017–2018 and among those over 60 was up to 74.5%. However, unlike hypertension in middle-aged population, in which both systolic blood pressure (SBP) and diastolic blood pressure (DBP) are elevated, hypertension in the older population is predominantly characterized by isolated systolic blood pressure elevation, and the pathogenesis is aortic stiffening.^31^ A paper published in 1997 from the prestigious Framingham Heart Study noted that SBP rises and DBP falls with age in people over 60 years old.^32^ Stiffening of the aorta reduces its cushioning capacity thereby elevating SBP, and the increased systolic burden on the heart impacts left ventricular ejection power, subsequently decreasing DBP.

Persistent high pressure of blood flow against artery walls is one of the main risks of arterial stiffness, while stiffened arteries can also be a cause of high SBP. On the one hand, pulse pressure impacts the arterial wall repeatedly causing elastin damage^33^; on the other hand, inflammation induces endothelial injury leading to the accumulation of lipids and others in the arterial wall, accelerating the process of arteries hardening.^34^ A longitudinal meta-analysis study found that patients with hypertension had significantly higher rates of cardiovascular events and mortality than the general population, as well as higher aortic PWV values.^35^ Monitoring arterial stiffness in older people seems to be useful in the management of hypertension and cardiovascular diseases.

### Joint disorder

Inflammatory joint diseases (IJD) are the most common type of joint disease involving rheumatoid arthritis (RA), ankylosing spondylitis (AS), and psoriatic arthritis (PsA). Besides joint symptoms, cardiovascular complications are IJD’s most frequent extra-articular manifestations.^36,37^ IHD and congestive heart failure secondary to coronary atherosclerosis are also major causes of death in patients with IJD.^38^ A meta-analysis found that patients with RA have a 48% higher risk of CVDs than the general population.^39^ Also, numerous patients diagnosed with PsA have a >10% risk of developing CVD within 10 years of the onset, yet these risks are underestimated by the traditional Framingham Risk Score.^40^ Aging as a primary risk factor for CVD risk also has a considerable impact on the risk of CVD in patients with IJD. A population-based cohort study of 563 patients with RA but no prior CVD followed up for a mean of 8.2 years revealed that the effect of age on CVD risk increased exponentially in patients with seropositive RA, whereas it was not significant in seronegative patients.^41^ Therefore, focusing on the risk of cardiovascular complications is essential for the treatment of patients with IJD.

### Esophageal disorder

Gastro-esophageal reflux disease (GERD) is a condition due to the reflux of stomach contents into the esophagus. Continued progression of GERD can lead to a range of esophageal disorders including esophageal stricture, Barrett esophagus, and esophageal adenocarcinoma. One cross-sectional study found that high-fat diets were positively associated with GERD risk, while high-fiber diets have been associated with a lower risk of GERD.^42^ High-fat eating habit damages the cardiovascular system likewise.^43^ Although there is no direct evidence that GERD is linked to a higher incidence of cardiovascular events, they share the same risk factor.

### Hyperthyroidism or thyrotoxicosis

Hyperthyroidism is a common endocrine disease, impacting at least 2.5% of adults all over the world, characterized by hypermetabolism and multiple organ dysfunction.^44^ Thyroid function is already known to have a strong and profound influence on the cardiovascular system; receptors for thyroid hormone are found in both vascular and myocardial endothelial tissues, and minute changes in circulating thyroid hormones concentrations can cause cardiovascular system effects. It was reported that thyroid hormones could directly target vascular smooth muscle cells, decreasing systemic resistance by reducing vascular tone and causing remodeling of small arteries.^45^ A study has found that l-thyroxine improves arterial endothelial function and reduces cardiovascular risk in patients with subclinical hypothyroidism,^46^ yet some other cohort studies have found that hyperthyroidism has a detrimental effect on arterial stiffness.^47^ The results that hyperthyroidism can reduce arterial stiffness will sound against common sense, as a connection between hyperthyroidism (even subclinical) and the risk of ventricular fibrillation has been widely reported.^48^

However, at the genomic level, thyroid hormone target genes are involved in different cellular pathways such as gluconeogenesis, insulin signaling, and lipogenesis.^49^ This implies that hyperthyroidism seemingly affects the cardiovascular system by multiple mechanisms, including dyslipidemia, hypertension, and myocardial dysfunction. As hypertension damages artery walls,^31^ prolonged and severe hyperthyroidism may increase arterial stiffness due to excessive arterial pressure, which increases the risk of cardiovascular complications. From a recent review, ultrasound images indicated that short-term hyperthyroidism enhances the left ventricle both in systolic and diastolic function, showing positive effects on the cardiovascular system.^50^ Still, in the long term, excess thyroid hormone seems to increase not only cardiac output but also cardiovascular mortality and morbidity.

### Bowel problem

Inflammatory bowel disease (IBD) is a chronic inflammatory disorder of the bowel caused by a combination of environmental, immunological, and genetic factors, typically ulcerative colitis and Crohn’s disease. Patients with IBD have a lower prevalence of obesity, dyslipidemia, and body mass index, which are three acknowledged cardiovascular risk factors, due to malabsorption.^51^ However, observational studies have found that patients with IBD appear to have a higher incidence of coronary heart disease, myocardial infarction (MI), and stroke;^52^ this is so-called the IBD paradox.^53^ Chronic inflammation has been suggested to be responsible for the increased cardiovascular risk, so further research is necessary to investigate whether control inflammation therapy would reduce the risk of CVD in patients with IBD.

### Health management advice for the aged population

The following are our recommendations for the health management of older people according to the conclusions of this study: (1) in patients with liver disease, gallbladder disease, or other digestive disorders, regular testing of ASI provides early assessment of cardiovascular event risk; (2) patients with hypertension should strictly control blood pressure levels in order to prevent arterial stiffening at an early stage; (3) for those with no history of digestive or hypertension but high ASI values, regular checkups as well as healthy diet and exercise are recommended; and (4) routine arterial stiffness screening is recommended for middle-aged and elderly populations and monitor the extent to which ASI increases with age. Prevention and intervention of diseases at an early stage may be possible through these measures.

**FIG. 3.**
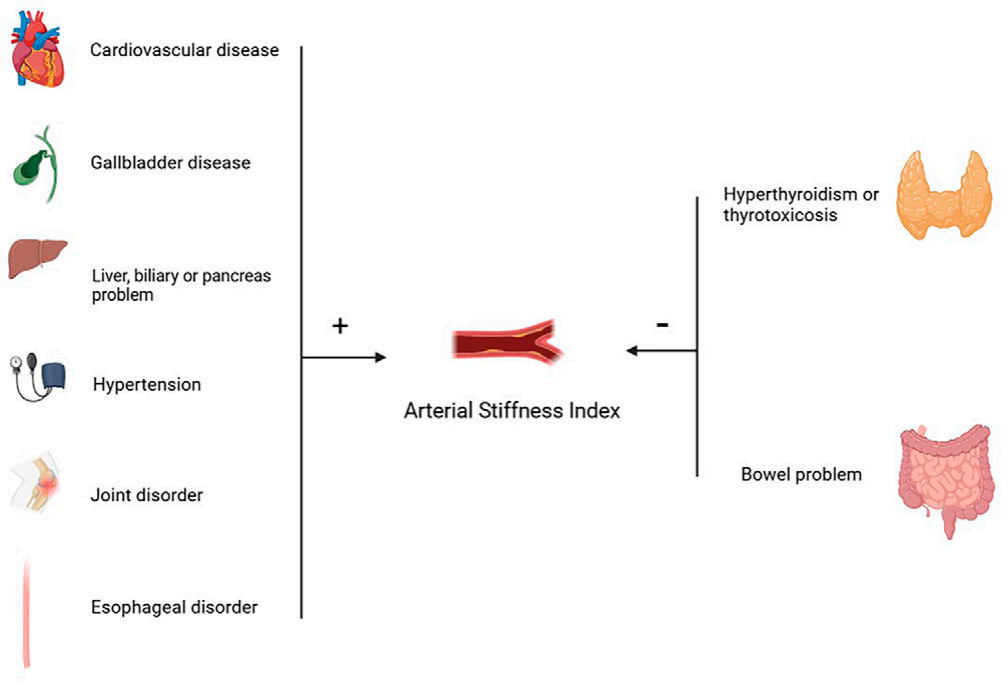
Association between ASI and eight age-related diseases.

In conclusion, using MR analyses, we identified at least eight age-related diseases that are causally associated with ASI (Fig. 3). As a simple, easy-to-use, and inexpensive measure of arterial stiffness, ASI promises to be an effective indicator of health screening in the older population. However, more clinical observational studies are needed in the future to confirm this causal relationship and include other ethnic populations.

## Data Availability

All GWAS data used in this study were obtained from published studies and open-access databases online (https://gwas.mrcieu.ac.uk).
