# Supplementary material for: Association Between Arterial Stiffness Index and Age-Related Diseases: A Mendelian Randomization Study
Source: Rejuvenation Res. 2025 Jan 28;28(1):9–16. doi: 10.1089/rej.2024.0041 (PMC11844224; doi:10.1089/rej.2024.0041)
Supplement: Supplementary Figure S4 [file rej.2024.0041_supp_figs4.pdf]

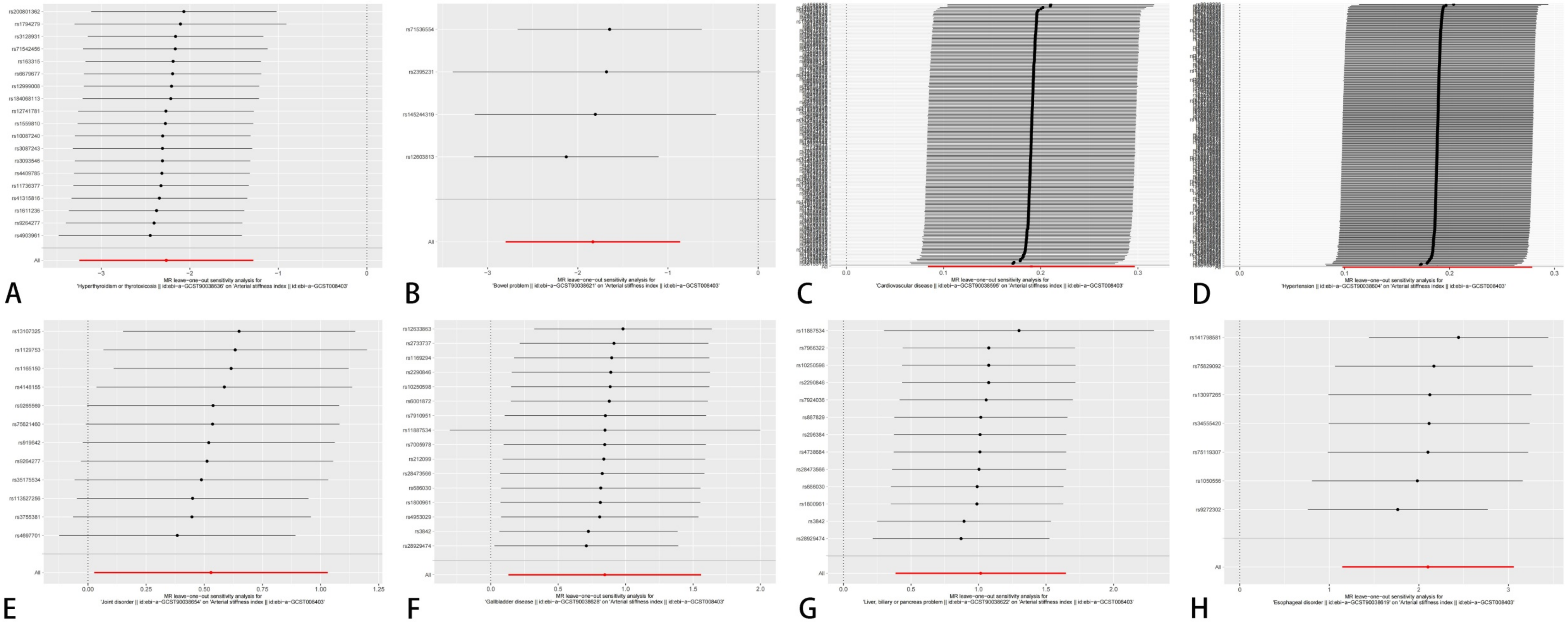

Figure S4. Leave-one-out sensitivity analysis of the association of arterial stiffness index and age-related diseases. (A) Hyperthyroidism or thyrotoxicosis; (B) Bowel problem; (C) Cardiovascular disease; (D) Hypertension; (E) Joint disorder; (F) Gallbladder disease; (G) Liver, biliary or pancreas problem; (H) Esophageal disorder.
