# Supplementary material for: Association Between Arterial Stiffness Index and Age-Related Diseases: A Mendelian Randomization Study
Source: Rejuvenation Res. 2025 Jan 28;28(1):9–16. doi: 10.1089/rej.2024.0041 (PMC11844224; doi:10.1089/rej.2024.0041)
Supplement: Supplementary Table S1 [file rej.2024.0041_supp_tables1.pdf]

Table S1. The instrumental variables SNPs for age-related diseases.

Cardiovascular disease

	SNP	effect allele	other allele	beta	EAF	chr	p-value
1	rs10059884	A	C	0.0101886	0.59623	5	1.69981E-28
2	rs10076730	C	T	-0.010225	0.375211	5	7.00003E-29
3	rs10100333	T	C	-0.006291	0.611547	8	2.19989E-11
4	rs10179344	A	T	-0.007917	0.11853	2	7.00003E-09
5	rs10213458	G	A	-0.005374	0.683775	4	8.99995E-09
6	rs10406499	C	T	-0.005722	0.712562	19	2.59998E-08
7	rs10500326	T	G	-0.00732	0.235237	16	1.50003E-12
8	rs1055129	G	A	-0.006657	0.304027	17	3.50026E-11
9	rs10622246	ATTTT	A	-0.00531	0.488572	7	4.60002E-09
10	rs1065853	T	G	-0.027209	0.080016	19	2.80027E-61
11	rs10776752	T	G	0.0122135	0.073969	1	4.90004E-13
12	rs10875713	T	A	0.0063739	0.211404	12	3.29997E-09
13	rs10876531	C	A	-0.008205	0.291282	12	2.99985E-16
14	rs10931284	T	C	0.0053334	0.628776	2	1.29999E-08
15	rs10966580	C	G	-0.006659	0.163428	9	4.20001E-08
16	rs11130157	T	G	0.0078953	0.655373	3	2.90001E-16
17	rs111663960	G	A	0.0093454	0.120482	6	5.50047E-12
18	rs112767262	T	C	0.0066525	0.188051	16	9.59997E-09
19	rs112977440	C	G	-0.00802	0.151559	2	2E-10
20	rs114165349	C	G	0.0199395	0.022281	1	8.10028E-11
21	rs11428568	AC	A	-0.006746	0.430937	16	7.50067E-14
22	rs11429307	GT	G	0.0094996	0.189098	5	2.09991E-16
23	rs11591147	T	G	-0.024579	0.016707	1	2.70023E-12
24	rs11688682	C	G	-0.006024	0.265463	2	8.60003E-09
25	rs1169294	A	G	0.0065908	0.30948	12	4.60045E-11
26	rs11724647	A	T	-0.010147	0.181422	4	1.80011E-18
27	rs11774829	A	T	-0.009036	0.096827	8	4.30002E-09
28	rs11943964	C	T	0.0057436	0.265751	4	9.20005E-09
29	rs11947277	T	C	0.0060656	0.256932	4	3.79997E-09
30	rs12116494	T	G	0.0052852	0.354348	1	0.000000016
31	rs12220375	C	T	-0.016082	0.080402	10	3.69999E-22
32	rs12258967	G	C	-0.009944	0.296214	10	3.80014E-24
33	rs12270515	A	G	-0.006704	0.214904	11	8E-10
34	rs12358504	G	A	0.0066248	0.207825	10	6.80002E-09
35	rs12368309	A	G	0.0079502	0.114978	12	1.40001E-08
36	rs12442901	G	A	-0.01116	0.731283	15	5.30029E-28
37	rs12446903	G	A	0.0074336	0.783858	16	1.29987E-11
38	rs12509595	C	T	0.0178671	0.287816	4	1.80011E-72
39	rs12627514	G	C	0.0070077	0.281587	21	2.90001E-12
40	rs12740374	T	G	-0.010844	0.221701	1	2.70023E-24

41	rs12747145	A	G	0.0061577	0.208905	1	2.90001E-08
42	rs1275988	T	C	-0.009996	0.603914	2	1.69981E-27
43	rs12762222	C	T	0.0225732	0.018847	10	2.90001E-11
44	rs12929303	A	G	0.0061056	0.52426	16	6.79986E-12
45	rs12978472	G	C	-0.018339	0.127229	19	6.29941E-42
46	rs13112725	C	G	0.008998	0.755169	4	3.90032E-18
47	rs13124618	T	C	-0.006045	0.653697	4	3.40001E-10
48	rs13128180	T	G	-0.005742	0.30837	4	6.1E-09
49	rs13221038	A	G	-0.005	0.498628	7	0.000000025
50	rs13286836	A	G	0.0104962	0.070329	9	3.69999E-09
51	rs13358657	G	A	0.0089738	0.130729	5	1E-11
52	rs1367117	A	G	0.0069512	0.32593	2	2.99985E-13
53	rs139114831	AG	A	0.0106376	0.084223	19	1.29999E-10
54	rs140435719	T	C	0.0054981	0.432735	8	1.29999E-09
55	rs1407588	A	G	0.0066579	0.415493	15	1.29987E-13
56	rs142158911	A	G	-0.015987	0.116141	19	3.29989E-30
57	rs144122622	C	G	-0.007718	0.161821	7	2.99999E-10
58	rs145153053	G	A	0.0088274	0.164684	17	2.90001E-13
59	rs146718647	T	C	0.0274182	0.016797	1	6.4003E-15
60	rs150305881	T	C	-0.005515	0.463885	5	5.30005E-09
61	rs1537370	T	C	0.0069584	0.490462	9	2.80027E-15
62	rs1547950	C	T	0.0052212	0.46511	3	1.40001E-08
63	rs1744349	G	A	0.0088888	0.263003	5	1.59993E-18
64	rs17677603	G	A	0.0068535	0.400279	5	2.29985E-13
65	rs1801253	C	G	0.010551	0.732637	10	2.60016E-25
66	rs1883711	C	G	0.0185934	0.030229	20	3.69999E-12
67	rs1886598	G	C	-0.006577	0.705076	10	4.40048E-11
68	rs1887320	A	G	0.0090305	0.476223	20	4.40048E-24
69	rs1894692	A	G	-0.018181	0.979028	1	0.000000012
70	rs1913657	T	C	0.0074645	0.646387	2	3.80014E-16
71	rs1962094	A	T	0.0053597	0.448943	10	2.99999E-09
72	rs2003943	G	C	0.0053852	0.361609	1	1.09999E-08
73	rs2004776	T	C	0.0084886	0.249337	1	3.50026E-16
74	rs2014590	T	C	-0.006145	0.487851	3	7.70016E-12
75	rs2078339	G	A	-0.007246	0.27628	12	4.90004E-13
76	rs2208589	G	A	0.0066413	0.775201	20	8.19993E-10
77	rs2209042	T	A	-0.006862	0.296745	6	2.49977E-12
78	rs2236295	T	G	-0.007696	0.39382	10	1.50003E-16
79	rs2306363	T	G	-0.008537	0.204574	11	4.19952E-15
80	rs2306527	T	C	-0.005789	0.515843	17	2.19999E-10
81	rs2400542	G	C	-0.005783	0.518578	8	2E-10
82	rs2412652	T	C	0.0056284	0.312803	15	4.70002E-09
83	rs2513995	G	C	-0.005448	0.273671	11	4.39997E-08
84	rs2521501	T	A	0.0116663	0.317606	15	9.30037E-33

85	rs2569882	C	T	-0.005283	0.444729	6	7.69999E-09
86	rs2643826	T	C	0.0081564	0.458311	3	4.40048E-19
87	rs2681492	C	T	-0.012429	0.175752	12	2.39994E-26
88	rs268263	A	T	0.0087447	0.742333	2	2.60016E-17
89	rs2760061	A	T	0.0057533	0.487367	1	1.2E-10
90	rs2823139	A	G	0.0061454	0.337739	21	1.09999E-10
91	rs28412876	T	G	0.0084482	0.367345	17	7.89951E-20
92	rs28601761	G	C	-0.010585	0.413046	8	1.80011E-30
93	rs28667801	T	A	0.0057669	0.405919	4	2.80001E-10
94	rs2886819	G	A	0.0056029	0.711886	2	1.29999E-08
95	rs300934	G	T	-0.006088	0.685974	4	1.5E-10
96	rs3208800	T	G	-0.004937	0.522544	22	2.30001E-08
97	rs34042070	G	C	0.0062735	0.19077	16	3.50002E-08
98	rs34592089	A	G	-0.018346	0.059408	4	5.30029E-22
99	rs34869093	G	A	0.0052669	0.36638	16	3.79997E-08
100	rs34881328	TC	T	0.0113932	0.127117	7	1.9002E-16
101	rs34937994	T	C	0.0070185	0.358506	11	6.79986E-14
102	rs35427	G	T	-0.009401	0.38557	12	2.99985E-23
103	rs35479618	A	G	0.0232335	0.016533	1	1.39991E-11
104	rs35797675	G	T	-0.007103	0.213476	7	8.69961E-11
105	rs36099535	G	A	-0.005032	0.391031	3	3.69999E-08
106	rs36174733	G	A	-0.007807	0.177337	12	4.19952E-11
107	rs3764769	T	C	-0.005668	0.257851	2	2.39999E-08
108	rs3785837	A	G	0.0061051	0.752062	17	0.000000015
109	rs3846663	T	C	0.0069967	0.376137	5	3.90032E-14
110	rs3918226	T	C	0.0248424	0.077846	7	5.60015E-49
111	rs4240321	G	T	-0.007605	0.866152	4	2.80001E-08
112	rs4380799	G	T	0.005963	0.386072	6	2.30001E-09
113	rs4548562	T	G	0.0081097	0.890891	10	1.89998E-08
114	rs4648624	T	G	0.0063051	0.418053	1	2.99985E-12
115	rs4775373	C	T	-0.005689	0.632064	15	2.69998E-09
116	rs4859684	T	C	-0.00576	0.635977	4	6.59994E-10
117	rs4910499	A	C	0.0077673	0.628593	11	1.99986E-17
118	rs4936682	G	T	0.0059469	0.274712	11	3.79997E-09
119	rs516246	T	C	0.0081997	0.501074	19	4.60045E-20
120	rs553108	G	A	0.0092645	0.586746	6	2.70023E-24
121	rs55851967	G	A	0.0058569	0.305168	4	2.19999E-09
122	rs55994889	CA	C	0.0104505	0.426458	6	3.40017E-30
123	rs56153133	G	A	-0.01525	0.161669	1	7.29962E-36
124	rs56330132	CTCA	C	0.0086138	0.187379	3	1.69981E-13
125	rs569550	G	T	0.0086085	0.385351	11	3.19963E-20
126	rs57866767	C	T	-0.007111	0.438051	10	2.39994E-15
127	rs58854324	C	T	0.0069621	0.166567	4	5.60003E-09
128	rs59400568	G	A	-0.007627	0.196942	9	1.50003E-11

129	rs604723	C	T	0.0129626	0.724885	11	1.29987E-37
130	rs6062343	A	G	-0.008414	0.434214	20	3.19963E-20
131	rs60772526	T	C	0.0135668	0.921267	7	8.80035E-16
132	rs6108168	A	C	-0.009398	0.26208	20	2.80027E-20
133	rs61772626	G	A	0.0084672	0.120839	1	1.2E-09
134	rs62434125	C	T	-0.015309	0.074905	6	1.59993E-19
135	rs62481856	A	G	0.008604	0.197249	7	8.69961E-15
136	rs645040	T	G	0.0075631	0.771723	3	2.49977E-12
137	rs6471502	A	G	-0.005982	0.528772	8	2.60016E-11
138	rs6504163	T	C	-0.009093	0.632755	17	1.80011E-22
139	rs6504213	C	T	0.0062337	0.595381	17	1.39991E-11
140	rs6740545	A	G	-0.008326	0.731757	2	1.29987E-16
141	rs6773653	A	G	-0.0115	0.083502	3	1.69981E-12
142	rs6865369	T	C	0.0067656	0.249529	5	1E-10
143	rs6903202	T	C	0.0051118	0.581734	6	4.39997E-08
144	rs6905288	A	G	0.0066412	0.573	6	8.19974E-14
145	rs6932812	G	C	-0.013364	0.076111	6	8.49963E-15
146	rs6938647	C	A	0.0064874	0.79029	6	6.59994E-09
147	rs696	T	C	0.0049653	0.370392	14	3.89996E-08
148	rs6961048	G	C	0.0092421	0.100663	7	1.2E-09
149	rs7015766	T	C	-0.009742	0.071126	8	3.50002E-08
150	rs702634	A	G	0.0063926	0.69657	5	6.00067E-11
151	rs71014329	GT	G	0.0054301	0.610012	1	1.09999E-08
152	rs71424153	C	G	-0.00594	0.245694	2	1.29999E-08
153	rs72683923	C	T	-0.01964	0.018908	14	1.29999E-09
154	rs72737758	T	C	0.0063072	0.429027	15	3.90032E-12
155	rs7278282	G	T	0.0052395	0.524374	21	1.89998E-09
156	rs72801474	A	G	-0.012064	0.088533	5	9.70063E-15
157	rs72831343	G	T	-0.01979	0.14019	10	5.50047E-53
158	rs7310615	G	C	-0.013255	0.535769	12	9.3994E-48
159	rs73126004	C	T	-0.005667	0.266019	12	2.99999E-08
160	rs73306876	G	A	0.0195289	0.118903	20	5.19996E-44
161	rs73563812	T	G	-0.008173	0.236983	8	1.10002E-14
162	rs73728279	T	G	0.0069441	0.274388	7	3.19963E-12
163	rs740047	T	C	0.0066872	0.800474	7	2.69998E-09
164	rs74439044	C	T	0.0097396	0.097654	17	1.89998E-10
165	rs74661587	G	A	0.0089533	0.137087	5	6.4998E-12
166	rs7585434	C	T	0.0053305	0.579975	2	1.29999E-08
167	rs76386877	C	T	0.0060806	0.417454	4	5.50047E-11
168	rs76452347	T	C	-0.009173	0.199028	9	4.60045E-15
169	rs76895963	G	T	-0.020964	0.020229	12	1.2E-09
170	rs76922886	A	G	0.0051678	0.321166	3	4.20001E-08
171	rs77301115	A	G	0.0194142	0.026422	19	3.80014E-12
172	rs7742789	T	C	0.0071467	0.302451	6	1.39991E-13

173	rs77924615	A	G	-0.01134	0.194052	16	6.90081E-24
174	rs780093	C	T	-0.008084	0.622045	2	1.39991E-17
175	rs78058190	A	G	0.0152867	0.049359	2	2.90001E-11
176	rs7903146	T	C	0.0057412	0.289723	10	5.49997E-09
177	rs7911644	T	C	0.0062374	0.33634	10	3.59998E-11
178	rs8084507	G	A	-0.00729	0.32746	18	5.79963E-14
179	rs9295128	T	G	0.0364449	0.016155	6	4.19952E-24
180	rs9317097	T	C	0.006532	0.814967	13	0.000000016
181	rs9368222	A	C	0.0064865	0.26142	6	1.79999E-10
182	rs9448580	G	C	0.0060922	0.491356	6	2.90001E-11
183	rs9549328	T	C	0.0064123	0.226821	13	1.29999E-09
184	rs964184	C	G	-0.01494	0.862505	11	2.80027E-31
185	rs972283	G	A	0.0080416	0.52148	7	7.19946E-19
186	rs9805764	T	C	0.0058623	0.291883	13	6.19998E-09
187	rs9836592	T	C	0.0054162	0.670487	3	0.000000021
188	rs9839000	G	A	0.0055843	0.330475	3	8.10009E-09
189	rs9844972	C	G	0.0128713	0.068617	3	1E-12
190	rs9848170	C	G	0.006288	0.594041	3	2.19989E-12

Gallbladder disease

	SNP	effect allele	other allele	beta	EAF	chr	p-value
1	rs10250598	G	A	-0.002383	0.209401	7	1.59993E-12
2	rs1169294	A	G	-0.00193	0.30948	12	4.10015E-11
3	rs11887534	C	G	0.0188194	0.065004	2	1E-200
4	rs12633863	A	G	-0.002377	0.543519	3	2.09991E-18
5	rs1800961	T	C	0.00563	0.030327	20	3.90032E-13
6	rs212099	A	T	-0.002951	0.161125	19	1.10002E-15
7	rs2290846	A	G	0.0021747	0.278092	4	3.90032E-13
8	rs2733737	C	T	-0.001744	0.623285	19	2.5E-10
9	rs28473566	A	G	-0.003697	0.163874	2	2.29985E-24
10	rs28929474	T	C	0.009403	0.01893	14	1.80011E-21
11	rs3842	C	T	-0.003138	0.143073	7	2.49977E-16
12	rs4953029	A	C	0.001924	0.244979	2	9.40005E-10
13	rs6001872	G	A	0.0015536	0.34843	22	2.90001E-08
14	rs679574	G	C	0.0019128	0.50109	19	2.39994E-12
15	rs686030	A	C	0.0025188	0.861793	9	6.09958E-11
16	rs7005978	G	A	-0.002126	0.665629	8	5.60015E-14
17	rs7910951	G	A	-0.001752	0.470285	10	8.30042E-11

Liver, biliary or pancreas problem

	SNP	effect allele	other allele	beta	EAF	chr	p-value
1	rs10250598	G	A	-0.002875	0.209401	7	1E-11
2	rs11887534	C	G	0.0191819	0.065004	2	7.4989E-176
3	rs1800961	T	C	0.0055684	0.030327	20	1.09999E-08
4	rs2290846	A	G	0.0024557	0.278092	4	5.90065E-11
5	rs28473566	A	G	-0.003423	0.163874	2	4.49987E-14

6	rs28929474	T	C	0.0115927	0.01893	14	5.70033E-21
7	rs296384	T	G	0.0025495	0.840823	19	0.000000032
8	rs3842	C	T	-0.003475	0.143073	7	4.10015E-13
9	rs4738684	G	A	-0.002045	0.66994	8	9.29994E-09
10	rs6774253	C	G	-0.001997	0.549711	3	4.70002E-09
11	rs686030	A	C	0.0027482	0.861793	9	1.40001E-08
12	rs7924036	T	G	0.0021888	0.501281	10	7.39946E-11
13	rs7966322	T	C	0.0020136	0.541091	12	2.69998E-08
14	rs887829	T	C	0.002298	0.319694	2	1.40001E-10

Hypertension

	SNP	effect allele	other allele	beta	EAF	chr	p-value
1	rs10059884	A	C	0.0129924	0.59623	5	1E-52
2	rs10076730	C	T	-0.011249	0.375211	5	2.49977E-39
3	rs10091532	A	C	-0.005963	0.41255	8	5.19996E-13
4	rs10164193	G	T	0.0092796	0.080216	18	2.99999E-09
5	rs10217559	T	C	0.0052701	0.678949	9	4.70002E-09
6	rs10224210	C	T	0.0084698	0.274673	7	4.70002E-20
7	rs10409243	T	C	-0.005593	0.593828	19	6.89922E-11
8	rs1047891	A	C	-0.005562	0.315455	2	4.60002E-10
9	rs1048070	C	T	0.0048095	0.530274	9	2.80001E-08
10	rs10500326	T	G	-0.008715	0.235237	16	5.90065E-20
11	rs10622246	ATTTT	A	-0.004738	0.488572	7	4.39997E-08
12	rs1065853	T	G	-0.011581	0.080016	19	3.50026E-14
13	rs10660539	CCTT	C	0.0054327	0.704586	1	2.99999E-09
14	rs10771960	T	C	0.0048539	0.339511	12	2.90001E-08
15	rs10776752	T	G	0.0186727	0.073969	1	4.19952E-32
16	rs10832586	C	A	0.010998	0.201177	11	5.79963E-27
17	rs10883543	T	G	0.0107306	0.892464	10	1.39991E-15
18	rs10943595	G	C	0.0062019	0.37458	6	1.20005E-12
19	rs10980408	C	T	0.0172267	0.036993	9	1.99986E-15
20	rs11006778	G	A	0.0049193	0.454373	10	3.69999E-09
21	rs11072508	T	C	-0.012178	0.646781	15	9.60064E-43
22	rs11112548	T	A	-0.01149	0.043821	12	2.59998E-08
23	rs11125883	C	A	-0.005663	0.361561	2	9.3994E-11
24	rs111896658	T	C	-0.009245	0.107826	16	3.69999E-12
25	rs11191559	T	C	-0.019608	0.081365	10	8.30042E-38
26	rs112035922	T	C	0.006352	0.239151	13	2.39994E-11
27	rs11249244	A	G	0.0067128	0.611172	1	6.00067E-15
28	rs112684153	C	T	-0.009837	0.07151	13	7.19996E-09
29	rs11429307	GT	G	0.0075628	0.189098	5	7.19946E-13
30	rs11441170	CT	C	-0.00952	0.300718	12	1.9002E-25
31	rs11454754	CT	C	0.0054754	0.634878	3	0.000000032
32	rs116511141	A	G	0.0134596	0.033077	3	0.00000001

33	rs11677903	T	C	-0.005505	0.208155	2	4.49997E-08
34	rs11688682	C	G	-0.006661	0.265463	2	8.00018E-12
35	rs117464403	A	G	0.0203903	0.018508	10	5.30029E-11
36	rs11771259	G	C	0.0098185	0.116445	7	5.60015E-14
37	rs11776122	G	A	0.0059436	0.199469	8	1.09999E-08
38	rs117777118	A	G	-0.012287	0.037554	18	4.79999E-08
39	rs11990607	G	A	-0.006747	0.170664	8	1.7E-09
40	rs12035750	C	T	0.0052855	0.372778	1	9.80009E-10
41	rs12114418	G	A	0.0060954	0.237412	8	8.30004E-10
42	rs12142296	G	T	0.0073714	0.134092	1	2.30001E-09
43	rs12237112	T	C	-0.005817	0.198511	9	3.40001E-08
44	rs12258967	G	C	-0.011794	0.296214	10	1.69981E-37
45	rs12368309	A	G	0.0084325	0.114978	12	1.40001E-10
46	rs12500686	G	A	-0.005355	0.287024	4	0.000000005
47	rs12509595	C	T	0.0217129	0.287816	4	1.9011E-124
48	rs12535256	C	T	0.0047349	0.441882	7	0.000000015
49	rs1275988	T	C	-0.014078	0.603914	2	2.29985E-61
50	rs12799296	A	G	0.008053	0.09363	11	7.49998E-09
51	rs12906125	A	G	0.0127158	0.321873	15	1.10002E-45
52	rs12906962	C	T	0.0067136	0.326499	15	3.29989E-14
53	rs1290933	A	C	-0.006035	0.679954	4	1.80011E-11
54	rs12911761	T	C	0.0048724	0.418175	15	0.000000016
55	rs12934751	C	T	0.0064329	0.536285	16	9.8992E-15
56	rs12978472	G	C	-0.020881	0.127229	19	5.90065E-62
57	rs13107325	T	C	-0.019887	0.07186	4	1.59993E-34
58	rs1317742	T	C	0.0069046	0.139752	11	4.90004E-09
59	rs1331860	T	G	0.0051903	0.356851	1	2.80001E-09
60	rs13358657	G	A	0.0092523	0.130729	5	2.09991E-14
61	rs139114831	AG	A	0.0087978	0.084223	19	7.00003E-09
62	rs139553812	TC	T	-0.007465	0.239177	2	2.60016E-15
63	rs1422278	T	G	0.00912	0.136272	5	2.19989E-14
64	rs142849507	T	C	0.0217307	0.016421	16	1.2E-09
65	rs143274148	T	C	0.0152713	0.03323	17	1.09999E-10
66	rs1436138	G	A	-0.00747	0.365272	17	1.10002E-17
67	rs145153053	G	A	0.0091578	0.164684	17	7.39946E-17
68	rs146718647	T	C	0.0296713	0.016797	1	6.4998E-20
69	rs150305881	T	C	-0.007401	0.463885	5	2.70023E-17
70	rs1599231	C	A	0.0061604	0.595228	3	2.99985E-13
71	rs169080	C	T	-0.005415	0.640273	19	5.69994E-10
72	rs17073834	C	G	0.0068873	0.123872	13	4.30002E-08
73	rs17099139	G	C	-0.005636	0.27256	10	2.69998E-09
74	rs17264894	A	T	-0.005381	0.425649	2	8.99912E-11
75	rs1741288	A	G	0.0048237	0.639585	20	2.69998E-08
76	rs17637472	A	G	0.0083426	0.386664	17	1.10002E-22

77	rs17677603	G	A	0.007337	0.400279	5	4.40048E-17
78	rs17826049	T	G	-0.011507	0.043799	7	2.59998E-08
79	rs1801253	C	G	0.0121583	0.732637	10	2.09991E-38
80	rs184863984	C	G	0.0123282	0.044928	7	2.30001E-09
81	rs1862043	C	G	-0.008169	0.636057	12	8.30042E-22
82	rs1886598	G	C	-0.006402	0.705076	10	6.29941E-12
83	rs1887320	A	G	0.0109438	0.476223	20	5.50047E-40
84	rs1923031	C	T	-0.007169	0.607755	20	1.99986E-16
85	rs193443	A	G	0.0051608	0.471327	14	1.5E-10
86	rs1981909	C	T	-0.005088	0.441302	2	4.39997E-09
87	rs198851	G	T	-0.012568	0.854083	6	1.39991E-27
88	rs1999996	G	A	0.0050317	0.447171	1	2.59998E-09
89	rs200009640	T	TCTCTC	0.0060181	0.203461	1	2.19999E-09
90	rs2014590	T	C	-0.006986	0.487851	3	2.99985E-17
91	rs2046645	G	C	-0.005499	0.436266	4	1.80011E-11
92	rs2071286	T	C	0.0101183	0.179635	6	6.90081E-21
93	rs2078339	G	A	-0.008125	0.27628	12	9.3994E-19
94	rs2105092	A	G	-0.007297	0.29745	6	1.20005E-15
95	rs2236295	T	G	-0.007444	0.39382	10	2.99985E-18
96	rs2242338	C	A	-0.011704	0.075813	3	6.59933E-14
97	rs2246445	G	T	0.0045558	0.597196	6	0.000000012
98	rs2251828	A	G	0.0062212	0.755937	6	5.30029E-11
99	rs2293252	T	C	-0.005359	0.654446	3	1.7E-09
100	rs2298359	C	T	-0.011843	0.064363	21	4.00037E-12
101	rs2306363	T	G	-0.008685	0.204574	11	1.10002E-17
102	rs2455357	G	A	-0.006894	0.70998	5	7.59976E-12
103	rs2460448	A	G	-0.008358	0.435511	16	7.10068E-23
104	rs2493296	T	C	0.0079618	0.137014	1	8.69961E-12
105	rs2527614	G	A	-0.006395	0.533771	1	4.40048E-15
106	rs2551976	C	T	-0.004943	0.480444	2	6.69993E-10
107	rs2569882	C	T	-0.005424	0.444729	6	1.6E-10
108	rs2581462	T	G	-0.004593	0.449886	15	4.79999E-08
109	rs2643826	T	C	0.0102905	0.458311	3	6.79986E-35
110	rs2681492	C	T	-0.014788	0.175752	12	8.4004E-42
111	rs268263	A	T	0.0085551	0.742333	2	3.19963E-19
112	rs2759309	A	G	0.0068862	0.451634	15	6.90081E-17
113	rs27687	C	T	0.0063675	0.267132	5	6.70039E-12
114	rs2823139	A	G	0.0060896	0.337739	21	5.10035E-12
115	rs28381984	T	C	0.0047218	0.494786	2	3.29997E-08
116	rs28394055	T	C	-0.006249	0.528854	8	1.39991E-13
117	rs28515660	C	T	-0.006188	0.259093	5	1E-10
118	rs28590346	T	A	0.0058847	0.345327	16	2.09991E-12
119	rs28667801	T	A	0.0071184	0.405919	4	1.10002E-16
120	rs28730491	C	G	-0.004978	0.336268	4	2.99999E-08

121	rs2921965	C	T	-0.005249	0.290767	10	0.000000017
122	rs2972147	C	T	0.0051878	0.651284	2	9.80009E-10
123	rs3093733	CA	C	-0.00737	0.282523	12	8.99912E-16
124	rs3117738	C	T	0.0095661	0.267182	5	6.20012E-25
125	rs3208800	T	G	-0.005308	0.522544	22	1.09999E-10
126	rs34344953	C	T	0.0055864	0.356274	7	2.60016E-11
127	rs35100507	AGT	A	0.005632	0.277873	13	3.50002E-09
128	rs35444	G	A	-0.010569	0.383264	12	6.70039E-35
129	rs35479618	A	G	0.0269047	0.016533	1	1.80011E-17
130	rs35769913	T	C	-0.005865	0.404451	7	1.20005E-11
131	rs35783704	A	G	-0.011101	0.09689	8	4.79954E-15
132	rs35895091	G	A	0.0052487	0.698704	4	4.90004E-09
133	rs360153	C	T	0.0081153	0.575157	11	9.09913E-24
134	rs36163465	G	A	-0.005429	0.321281	8	0.000000016
135	rs36174733	G	A	-0.006661	0.177337	12	1.5E-09
136	rs3757394	T	C	0.0056856	0.623008	7	4.60045E-12
137	rs3759582	C	A	-0.00764	0.126937	14	3.2E-09
138	rs3764769	T	C	-0.006803	0.257851	2	2.39994E-13
139	rs3785837	A	G	0.0075877	0.752062	17	7.29962E-15
140	rs3803266	C	G	-0.00827	0.751347	13	9.8992E-18
141	rs3809060	T	G	0.0054209	0.389138	11	7.79992E-10
142	rs3863105	C	T	0.0050292	0.679326	3	6.29999E-09
143	rs3867466	C	A	0.0076467	0.277949	11	4.79954E-17
144	rs3918226	T	C	0.0261951	0.077846	7	7.8001E-63
145	rs4277405	T	C	-0.008755	0.625058	17	4.30031E-25
146	rs4335411	A	G	0.0063954	0.7674	1	3.09999E-10
147	rs4390812	A	G	-0.006138	0.508885	2	2.70023E-13
148	rs4392188	G	A	0.0076366	0.122948	2	2.99999E-09
149	rs4648624	T	G	0.0060989	0.418053	1	2.80027E-13
150	rs4682671	G	T	0.006733	0.590775	3	1E-15
151	rs473188	G	C	-0.008448	0.885545	11	1.2E-10
152	rs4752518	T	C	0.0075101	0.200345	10	2.90001E-12
153	rs4803195	T	G	-0.006859	0.175624	19	9.09997E-10
154	rs4805881	C	A	-0.005992	0.662233	19	1.80011E-11
155	rs4833588	G	A	-0.009274	0.871297	4	1.10002E-13
156	rs4842504	C	A	-0.004676	0.529243	12	5.30005E-09
157	rs488834	T	C	-0.007949	0.758521	1	2.80027E-16
158	rs4924588	G	A	0.0065328	0.355485	15	6.89922E-14
159	rs4980986	A	T	0.006566	0.559175	12	2.80027E-15
160	rs5020545	T	C	-0.005248	0.435734	4	4.90004E-10
161	rs532436	A	G	-0.008438	0.182661	9	1.10002E-15
162	rs55735727	T	A	-0.005417	0.267267	3	0.00000001
163	rs55994889	CA	C	0.0123249	0.426458	6	6.89922E-47
164	rs56153133	G	A	-0.017728	0.161669	1	1.59993E-55

165	rs569550	G	T	0.0117103	0.385351	11	7.10068E-42
166	rs57139556	G	A	-0.018811	0.076333	6	1.39991E-33
167	rs57158761	G	A	0.0052262	0.442106	3	5.50047E-11
168	rs57778433	A	G	-0.009477	0.081786	1	2E-10
169	rs57786342	A	G	0.0062025	0.20445	14	2.30001E-09
170	rs57866767	C	T	-0.009963	0.438051	10	3.29989E-33
171	rs581441	C	G	0.0050675	0.431166	18	1.09999E-08
172	rs5836558	ATTT	A	0.0048261	0.569643	2	0.000000025
173	rs58854324	C	T	0.006607	0.166567	4	2.39999E-09
174	rs59867374	A	G	0.0060366	0.592011	16	1.10002E-12
175	rs6026742	A	G	0.0195389	0.118989	20	6.20012E-51
176	rs6031431	G	A	0.0064218	0.459955	20	4.19952E-14
177	rs6033019	T	G	0.0224885	0.013285	20	7.49998E-10
178	rs604723	C	T	0.0141871	0.724885	11	7.39946E-53
179	rs60772526	T	C	0.0181223	0.921267	7	3.90032E-31
180	rs60984214	A	G	0.0054121	0.216511	12	0.00000002
181	rs6108168	A	C	-0.011185	0.26208	20	5.60015E-33
182	rs6108676	A	C	-0.005361	0.369675	20	9.69996E-10
183	rs61772592	G	A	0.008194	0.120567	1	1.09999E-10
184	rs62189015	C	T	-0.007533	0.099666	2	3.89996E-08
185	rs62271373	A	T	0.0134925	0.057817	3	1.80011E-13
186	rs62429779	A	G	0.0064626	0.150357	6	2.80001E-08
187	rs62460739	A	G	-0.005288	0.273082	7	5.69994E-09
188	rs62481856	A	G	0.0101412	0.197249	7	1.59993E-22
189	rs6271	T	C	-0.01273	0.070997	9	1.69981E-15
190	rs6441207	T	C	0.0049571	0.407275	3	3.2E-09
191	rs6445819	T	C	-0.005766	0.324825	3	8.60003E-11
192	rs6456010	T	A	0.011419	0.930721	6	2.19989E-11
193	rs6471502	A	G	-0.005732	0.528772	8	8.30042E-12
194	rs6504213	C	T	0.0051426	0.595381	17	1.7E-09
195	rs6568443	C	T	-0.005844	0.80889	6	6.29999E-09
196	rs6666703	T	C	-0.005144	0.286961	1	4.79999E-08
197	rs6768611	A	G	0.0076994	0.666495	3	2.09991E-18
198	rs6784520	A	C	-0.005269	0.41895	3	1.09999E-10
199	rs6800730	G	A	0.0086412	0.663214	3	3.29989E-22
200	rs68085857	T	C	0.0063482	0.229731	1	8.30042E-11
201	rs681343	T	C	0.0061731	0.501097	19	5.19996E-14
202	rs68192516	G	T	0.0057593	0.302104	1	1E-10
203	rs6841386	A	C	0.0083658	0.852984	4	2.19989E-13
204	rs6883793	C	A	-0.005103	0.442147	5	2.90001E-10
205	rs6905288	A	G	0.0054424	0.573	6	4.79954E-11
206	rs6934891	A	G	0.0055594	0.427298	6	2.39994E-11
207	rs696	T	C	0.0057886	0.370392	14	4.40048E-12
208	rs6961048	G	C	0.0114059	0.100663	7	2.60016E-16

209	rs702634	A	G	0.0060572	0.69657	5	1.29987E-11
210	rs7107372	T	C	0.0057325	0.262933	11	1.29999E-09
211	rs7118561	G	A	-0.007079	0.173505	11	2.99999E-10
212	rs7125196	C	T	-0.009979	0.120575	11	3.50026E-15
213	rs71382367	AGAT	A	0.0059891	0.4512	16	1.69981E-12
214	rs71475909	G	A	0.0105825	0.35299	11	4.70002E-33
215	rs7186298	T	C	-0.004781	0.43157	16	0.000000017
216	rs7232858	C	T	-0.00687	0.189308	18	1.40001E-10
217	rs7259285	A	G	0.0048771	0.449102	19	2.39999E-09
218	rs72683923	C	T	-0.019188	0.018908	14	1.09999E-10
219	rs72689147	T	G	-0.009933	0.181425	4	1.99986E-20
220	rs7276316	T	A	0.0055094	0.597926	21	1.9002E-11
221	rs72785660	G	A	-0.006188	0.150218	5	2.59998E-08
222	rs72801474	A	G	-0.009299	0.088533	5	1.79999E-10
223	rs72831343	G	T	-0.020622	0.14019	10	1.39991E-67
224	rs72838866	G	A	0.0091961	0.131634	6	2.49977E-13
225	rs72915163	T	C	0.0077444	0.257712	18	4.60045E-16
226	rs72971364	A	G	0.0078548	0.099023	18	4.60002E-08
227	rs73053851	G	C	-0.005426	0.258992	12	2.39999E-08
228	rs73099903	T	C	0.0094868	0.084881	12	2.90001E-10
229	rs7310615	G	C	-0.013302	0.535769	12	2.29985E-56
230	rs73126004	C	T	-0.005676	0.266019	12	0.000000002
231	rs740047	T	C	0.0073202	0.800474	7	3.19963E-12
232	rs7404754	T	C	-0.005772	0.574126	16	2.99985E-12
233	rs74439044	C	T	0.0121976	0.097654	17	4.19952E-18
234	rs7515635	C	T	-0.004855	0.538697	1	3.50002E-09
235	rs754064	T	C	-0.007128	0.148831	20	6.80002E-10
236	rs76452347	T	C	-0.010753	0.199028	9	3.59998E-23
237	rs7763350	C	A	0.008045	0.331133	6	4.90004E-20
238	rs778124	A	G	0.0051876	0.37539	1	1.6E-09
239	rs77924615	A	G	-0.013238	0.194052	16	3.19963E-36
240	rs78083376	T	G	-0.005576	0.247362	1	7.69999E-09
241	rs7826620	T	C	0.0060784	0.325054	8	4.60045E-12
242	rs7831859	C	T	-0.005744	0.431949	8	6.00067E-12
243	rs7838131	A	G	-0.006148	0.564456	8	1.39991E-13
244	rs7841408	G	A	-0.006011	0.348299	8	3.19963E-12
245	rs7844259	A	G	0.0058511	0.730581	8	4.90004E-10
246	rs79094191	C	T	0.0175324	0.037683	21	1.99986E-15
247	rs7911644	T	C	0.0081745	0.33634	10	6.20012E-21
248	rs79384779	T	C	0.0078536	0.148326	20	1.5E-10
249	rs79598313	T	C	0.0174486	0.022523	1	5.39995E-10
250	rs8073626	T	C	-0.005047	0.524678	17	9.20005E-10
251	rs8093196	T	G	0.0068251	0.66915	18	2.29985E-14
252	rs8131909	G	C	0.008563	0.125329	21	1.39991E-11

253	rs8243	A	C	-0.005782	0.625495	2	8.00018E-11
254	rs848309	C	T	0.0049803	0.575103	1	1.79999E-09
255	rs886629	C	A	-0.007307	0.684122	7	8.80035E-16
256	rs893929	A	G	-0.005845	0.450955	4	1.9002E-12
257	rs902450	A	G	-0.005703	0.720001	3	2.69998E-10
258	rs9268671	G	A	0.0063349	0.662242	6	6.29941E-13
259	rs9330353	A	T	0.0065194	0.426693	4	4.70002E-15
260	rs9399953	T	C	0.0053899	0.307175	6	1.29999E-08
261	rs943580	A	G	-0.008075	0.585591	1	8.4004E-22
262	rs9479072	T	C	-0.005527	0.582209	6	8.10028E-11
263	rs9506725	C	T	-0.006995	0.359383	13	5.10035E-16
264	rs951914	C	G	0.0086898	0.711986	8	1.20005E-21
265	rs9690885	T	C	-0.008257	0.191017	7	1.80011E-14
266	rs972283	G	A	0.007505	0.52148	7	1.29987E-19
267	rs9747806	A	G	-0.00489	0.513276	17	5.49997E-09
268	rs9751828	A	G	0.0056294	0.713322	2	4.39997E-09
269	rs990619	G	C	0.0060784	0.524638	4	6.89922E-13
270	rs9907987	C	T	0.0082997	0.9059	17	6.80002E-09
271	rs9917785	G	A	-0.004649	0.338253	3	2.80001E-08

Joint disorder

	SNP	effect allele	other allele	beta	EAF	chr	p-value
1	rs1129753	T	C	0.0100292	0.164412	6	1.50003E-26
2	rs113527256	TA	T	0.0049502	0.168656	15	0.000000021
3	rs1165150	T	C	0.0042305	0.550592	6	2.19999E-10
4	rs13107325	T	C	0.0080209	0.07186	4	3.50002E-10
5	rs35175534	C	A	0.0076725	0.137635	6	3.40017E-13
6	rs3755381	C	T	-0.004299	0.523596	2	7.00003E-11
7	rs4148155	G	A	0.0086336	0.112185	4	1.9002E-16
8	rs4697701	A	G	-0.005785	0.277933	4	4.60045E-15
9	rs75621460	A	G	0.0141798	0.026307	19	4E-10
10	rs919642	T	A	0.0044678	0.266855	9	4.90004E-09
11	rs9264277	C	T	0.0041897	0.634299	6	1.29999E-09
12	rs9265569	A	G	0.0041268	0.435287	6	6.69993E-09

Esophageal disorder

	SNP	effect allele	other allele	beta	EAF	chr	p-value
1	rs1050556	T	C	0.0040942	0.511415	6	1.20005E-15
2	rs13097265	A	G	0.0035099	0.282605	3	2.99999E-10
3	rs141798581	G	A	0.0091276	0.027726	8	3.79997E-09
4	rs34555420	T	G	-0.004985	0.094078	6	6.4E-09
5	rs34555420	T	G	-0.004985	0.094078	6	6.4E-09
6	rs75119307	T	C	0.0085247	0.028668	19	1.29999E-08
7	rs75829092	T	C	0.0075449	0.037006	8	1.79999E-08
8	rs9272302	T	C	-0.004228	0.162969	6	2.1E-09

Hyperthyroidism or thyrotoxicosis

	SNP	effect allele	other allele	beta	EAF	chr	p-value
1	rs10087240	T	C	0.0011336	0.458311	8	2.30001E-10
2	rs11736377	T	C	-0.001145	0.737388	4	1.40001E-08
3	rs12741781	G	T	0.0011479	0.328488	1	1.89998E-09
4	rs12999008	A	T	0.0045727	0.01272	2	2.69998E-08
5	rs1559810	A	C	0.0010799	0.406014	3	2.19999E-09
6	rs1611236	A	G	-0.001057	0.674823	6	1.79999E-09
7	rs163315	T	G	0.0014246	0.145897	5	2.30001E-08
8	rs1794279	T	G	0.0068843	0.120389	6	6.9024E-134
9	rs184068113	C	T	0.0016351	0.401785	6	1.69981E-13
10	rs200801362	C	T	0.0050729	0.157012	6	1.59993E-50
11	rs3087243	A	G	-0.001927	0.448193	2	8.99912E-27
12	rs3093546	A	G	-0.002373	0.045312	6	9.59997E-09
13	rs3128931	A	G	-0.001195	0.251339	6	2.99999E-09
14	rs41315816	C	T	-0.002449	0.061221	6	0.00000002
15	rs4409785	C	T	0.0014403	0.171039	11	1.09999E-09
16	rs4903961	G	C	0.002559	0.376258	14	1.39991E-43
17	rs6679677	A	C	0.0025648	0.096716	1	1.59993E-17
18	rs71542456	G	A	0.0041361	0.200752	6	1.10002E-47
19	rs9264277	C	T	0.0014018	0.634299	6	2.99985E-17

Bowel problem

	SNP	effect allele	other allele	beta	EAF	chr	p-value
1	rs113301125	C	G	-0.003017	0.527522	6	2.39999E-08
2	rs12603813	C	T	0.0034356	0.250277	17	3.79997E-09
3	rs145244319	A	G	0.0041681	0.45279	6	3.90032E-14
4	rs2395231	A	G	0.0089804	0.121165	6	4.19952E-31
5	rs71536554	C	T	0.0050352	0.169044	6	9.40005E-09

SNP, single nucleotide polymorphism; EAF, effective allele frequency; chr, chromosome.
