# Supplementary material for: Association Between Arterial Stiffness Index and Age-Related Diseases: A Mendelian Randomization Study
Source: Rejuvenation Res. 2025 Jan 28;28(1):9–16. doi: 10.1089/rej.2024.0041 (PMC11844224; doi:10.1089/rej.2024.0041)
Supplement: Supplementary Table S2 [file rej.2024.0041_supp_tables2.pdf]

Table S2. The instrumental variables SNPs for ASI.

	SNP	effect allele	other allele	beta	EAF	chr	p-value
1	rs1930290	C	T	0.0230174	0.447299	1	1.10E-08
2	rs1006923	C	T	-0.0293436	0.322594	2	5.30E-12
3	rs10840457	G	A	0.0235791	0.686309	11	3.40E-08
4	rs7331212	A	G	-0.0301338	0.262912	13	2.20E-11
