# Supplementary material for: Association Between Arterial Stiffness Index and Age-Related Diseases: A Mendelian Randomization Study
Source: Rejuvenation Res. 2025 Jan 28;28(1):9–16. doi: 10.1089/rej.2024.0041 (PMC11844224; doi:10.1089/rej.2024.0041)
Supplement: Supplementary Table S3 [file rej.2024.0041_supp_tables3.pdf]

Table S3. Full results of MR analysis.

Exposure	Outcome	IVW			MR-Egger			Weighted Median		
		β	SE	p	β	SE	p	β	SE	p
Cardiovascular disease	ASI	0.19	0.05	4.52E-04	0.22	0.15	0.13	0.18	0.07	8.11E-03
Gallbladder disease		0.85	0.36	0.02	1.31	0.51	0.02	0.84	0.40	0.04
Liver, biliary or pancreas problem		1.02	0.32	1.59E-03	1.21	0.46	0.02	0.86	0.40	0.03
Hypertension		0.19	0.05	4.43E-05	0.33	0.12	6.89E-03	0.20	0.06	8.12E-04
Joint disorder		0.53	0.26	0.04	-0.20	0.73	0.79	0.40	0.35	0.25
Esophageal disorder		2.10	0.49	1.68E-05	-0.05	1.56	9.77E-01	2.03	0.64	1.51E-03
Hyperthyroidism or thyrotoxicosis		-2.17	0.50	6.10E-06	-3.35	0.88	1.40E-03	-2.60	0.70	2.08E-04
Bowel problem		-1.83	0.49	2.05E-04	-2.86	1.41	0.18	-1.96	0.57	5.88E-04

IVW, inverse variance weighted; MR-Egger, Mendelian randomization with Egger regression; β, beta coefficient; SE, standard error; p, p-value.
