# Supplementary material for: Association Between Arterial Stiffness Index and Age-Related Diseases: A Mendelian Randomization Study
Source: Rejuvenation Res. 2025 Jan 28;28(1):9–16. doi: 10.1089/rej.2024.0041 (PMC11844224; doi:10.1089/rej.2024.0041)
Supplement: Supplementary Table S4 [file rej.2024.0041_supp_tables4.pdf]

Table S4. Results of reverse MR analysis.

Exposure	Outcome	IVW			MR-Egger			Weighted Median		
		β	SE	p	β	SE	p	β	SE	p
ASI	Cardiovascular disease	0.02	0.03	0.53	0.24	0.25	0.43	0.03	0.02	0.14
	Gallbladder disease	-5.04E-03	8.71E-03	0.56	-3.26E-03	0.09	0.97	-3.88E-03	7.63E-03	0.61
	Liver, biliary or pancreas problem	-0.01	0.01	0.34	0.03	0.11	0.83	-0.01	9.35E-03	0.13
	Hypertension	0.04	0.05	0.43	0.62	0.25	0.13	0.04	0.03	0.16
	Joint disorder	-0.03	0.02	0.26	0.18	0.18	0.42	-0.01	0.02	0.56
	Esophageal disorder	-8.00E-03	0.01	0.52	-0.05	0.12	0.72	-3.73E-04	0.01	0.98
	Hyperthyroidism or thyrotoxicosis	-5.36E-04	3.64E-03	0.88	0.03	0.03	0.38	5.44E-04	4.11E-03	0.89
	Bowel problem	4.98E-03	0.01	0.69	-0.07	0.11	0.61	3.02E-04	0.01	0.98
